# Analysis of the Use of shRNA in Atherosclerosis Therapy: Current Status

**DOI:** 10.3390/cells15212032

**Published:** 2026-11-07

**Authors:** Alexander V. Blagov, Anastasia O. Maksaeva, Tatiana I. Kovyanova

**Affiliations:** 1 Laboratory of Molecular Genetic Modeling of Inflammaging, Institute of General Pathology and Pathophysiology, 125315 Moscow, Russia; 2 Institute for Atherosclerosis Research, 121609 Moscow, Russia

**Keywords:** RNA interference, RNA, small interfering, atherosclerosis, genetic therapy, genetic vectors, Dependovirus, proprotein convertase 9, NLR family, pyrin domain-containing 3 protein

## Abstract

Atherosclerosis remains the principal pathological substrate of myocardial infarction, ischemic stroke and peripheral artery disease, and its progression is driven by an interlocking set of processes, including hepatic and vascular lipid metabolism, chronic vascular inflammation, macrophage foam cell formation and vascular smooth muscle cell (VSMC) proliferation, none of which is fully controlled by currently approved pharmacotherapy. Short hairpin RNA (shRNA) technology, in which a DNA template encoding a stem-loop transcript is delivered to a cell and processed intracellularly into small interfering RNAs (siRNAs) that trigger sequence-specific mRNA degradation, offers a route to durable, vector-encoded gene silencing that is mechanistically distinct from transiently acting synthetic siRNA or antisense oligonucleotides and that, although non-permanent at the level of the target gene, is not readily reversible at the level of the vector, which cannot easily be withdrawn once administered. This review analyzes the current status of shRNA-based strategies for atherosclerosis therapy. We summarize the molecular mechanism and vector design principles of shRNA-mediated RNA interference (RNAi); critically examine preclinical studies in which shRNA delivered by lentiviral or adeno-associated viral (AAV) vectors has been used to silence hepatic lipid-metabolism genes (PCSK9, ApoB,), the endothelial gene ANGPTL2, inflammasome and inflammatory mediators (NLRP3, TACE/ADAM17), and genes driving VSMC proliferation in restenosis models (connexin 43); we explicitly distinguish this direct shRNA evidence from complementary target-validation studies that use antisense oligonucleotides (e.g., ANGPTL4), synthetic siRNA, genetic knockout, or antimiR approaches (e.g., lncRNA AK136714, CD36, miR-33, P2X7R); and discuss the principal delivery vectors, their tissue tropism, and the toxicity arising from saturation of the endogenous microRNA-processing machinery. We further place shRNA in the context of competing and complementary RNA-based and gene-editing modalities, including clinically approved synthetic siRNA drugs (inclisiran, patisiran) and CRISPR-based gene editing of PCSK9 and ANGPTL3, both of which have now yielded human phase 1 data, to clarify why, despite two decades of robust preclinical proof-of-concept, no shRNA construct has yet reached clinical use in atherosclerosis; we further compare this with the clinical trajectory of vector-encoded shRNA in other indications, in which anti-*CCR5* and anti-HCV constructs have already been administered to patients in phase 1/2 trials, in order to establish that the limiting factor is not the hairpin itself but the risk–benefit case obtainable in a chronic, largely asymptomatic disease for which withdrawable and outcome-validated therapies already exist. We conclude that shRNA remains a mechanistically attractive but clinically immature modality whose translation will depend on vector engineering that limits off-target saturation of the RNAi pathway and achieves durable, cell type-restricted expression in the liver, endothelium, macrophages or vascular wall, and, equally, on the identification of an atherosclerosis indication—most plausibly a small, high-residual-risk or locally treatable population—in which durability outweighs the vector-level irreversibility, immunogenicity and manufacturing burden of a viral vector.

## 1. Introduction

**Figure 1:**
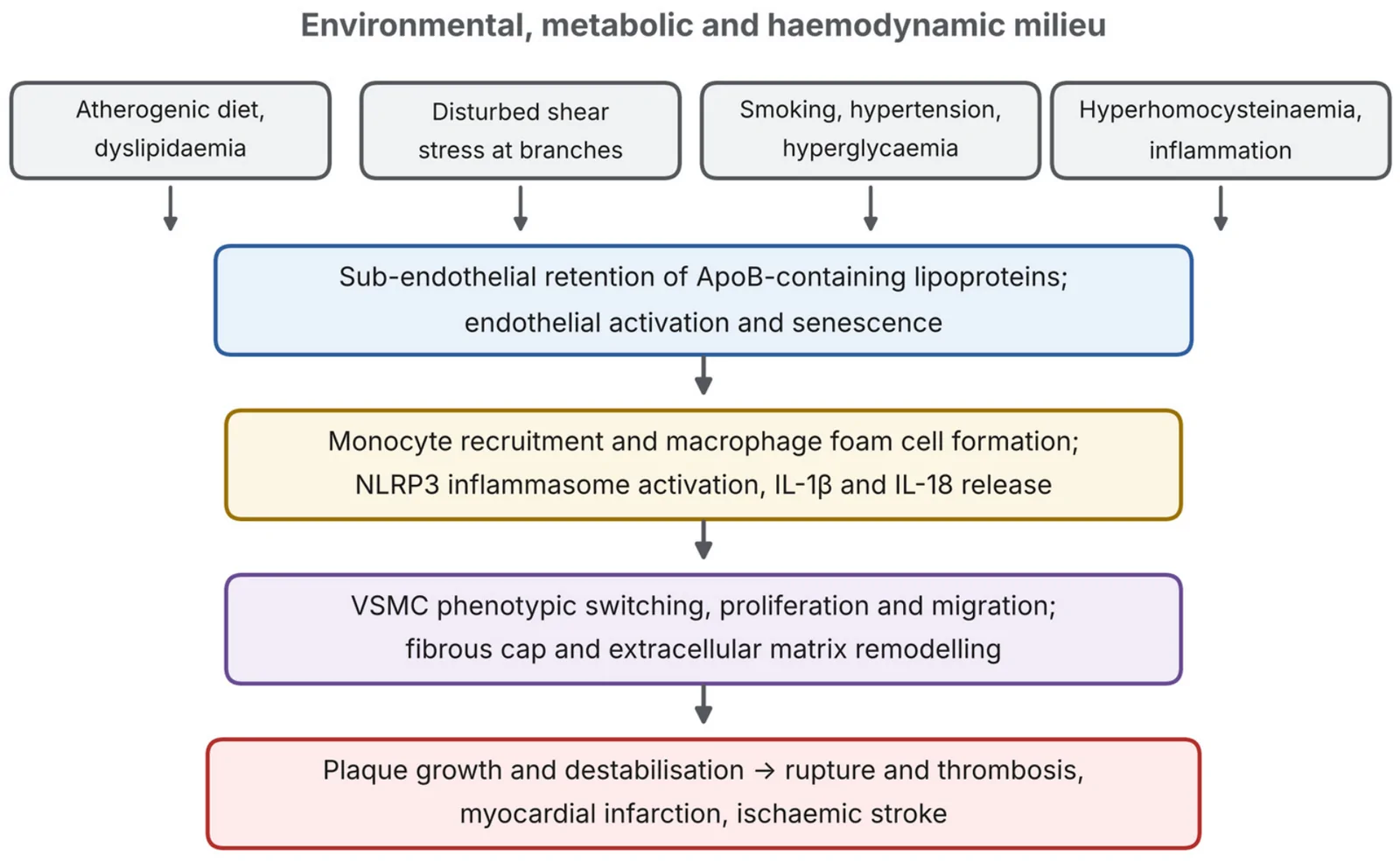
Environmental, metabolic and hemodynamic drivers of atherosclerotic plaque progression. Atherogenic diet and dyslipidemia, disturbed shear stress at arterial branches and bends, smoking, hypertension and hyperglycemia, and hyperhomocysteinemia with systemic inflammation converge on the arterial wall. These stimuli promote sub-endothelial retention of apolipoprotein B-containing lipoproteins together with endothelial activation and senescence; this is followed by monocyte recruitment and macrophage foam cell formation, with NLRP3 inflammasome activation and release of interleukin-1β (IL-1β) and IL-18; then by vascular smooth muscle cell (VSMC) phenotypic switching, proliferation and migration, with fibrous cap and extracellular matrix remodeling; and finally by plaque growth and destabilization, leading to rupture and thrombosis, myocardial infarction and ischemic stroke. Each stage supplies the gene targets addressed by the shRNA studies reviewed in Section 3, Section 4, Section 5, Section 6 and Section 7.

Atherosclerosis is a chronic disease of the arterial wall characterized by sub-endothelial retention of apolipoprotein B-containing lipoproteins, recruitment of monocytes that differentiate into lipid-laden macrophages, proliferation and phenotypic switching of VSMCs, and progressive fibrous and, in advanced lesions, calcific remodeling of the vessel wall, culminating in plaque rupture, thrombosis, and acute cardiovascular events [1]. The environmental, metabolic and hemodynamic context in which this sequence unfolds, and the successive cellular stages through which it progresses, are summarized in Figure 1. Statins, ezetimibe, and the more recently introduced proprotein convertase subtilisin/kexin type 9 (PCSK9) inhibitors have substantially reduced, but not eliminated, the residual cardiovascular risk associated with hyperlipidemia and vascular inflammation, as reflected in current lipid-management clinical practice guidelines [2], which has sustained a search for therapeutic modalities capable of durably silencing individual disease-driving genes rather than modulating their protein products pharmacologically.

RNA interference (RNAi) is an evolutionarily conserved post-transcriptional gene-silencing mechanism, first described in *Caenorhabditis elegans*, in which double-stranded RNA triggers sequence-specific degradation of homologous mRNA [3]. Synthetic siRNA duplexes were subsequently shown to elicit RNAi in mammalian cells without the non-specific interferon response triggered by long double-stranded RNA [4], but because synthetic siRNA is diluted with each cell division, several groups went on to develop vector-encoded short hairpin RNA (shRNA) as a self-renewing alternative; the resulting molecular distinction between siRNA and shRNA, including vector and promoter design, is detailed in Section 2.1 and Section 2.2 [5,6,7]. This vector-encoded, self-renewing property is what mechanistically distinguishes shRNA from synthetic siRNA and antisense oligonucleotides, and it is the reason shRNA has been pursued specifically for chronic diseases, such as atherosclerosis, in which years of sustained target suppression, rather than a single transient pulse, is likely to be required for clinical benefit.

**Figure 2:**
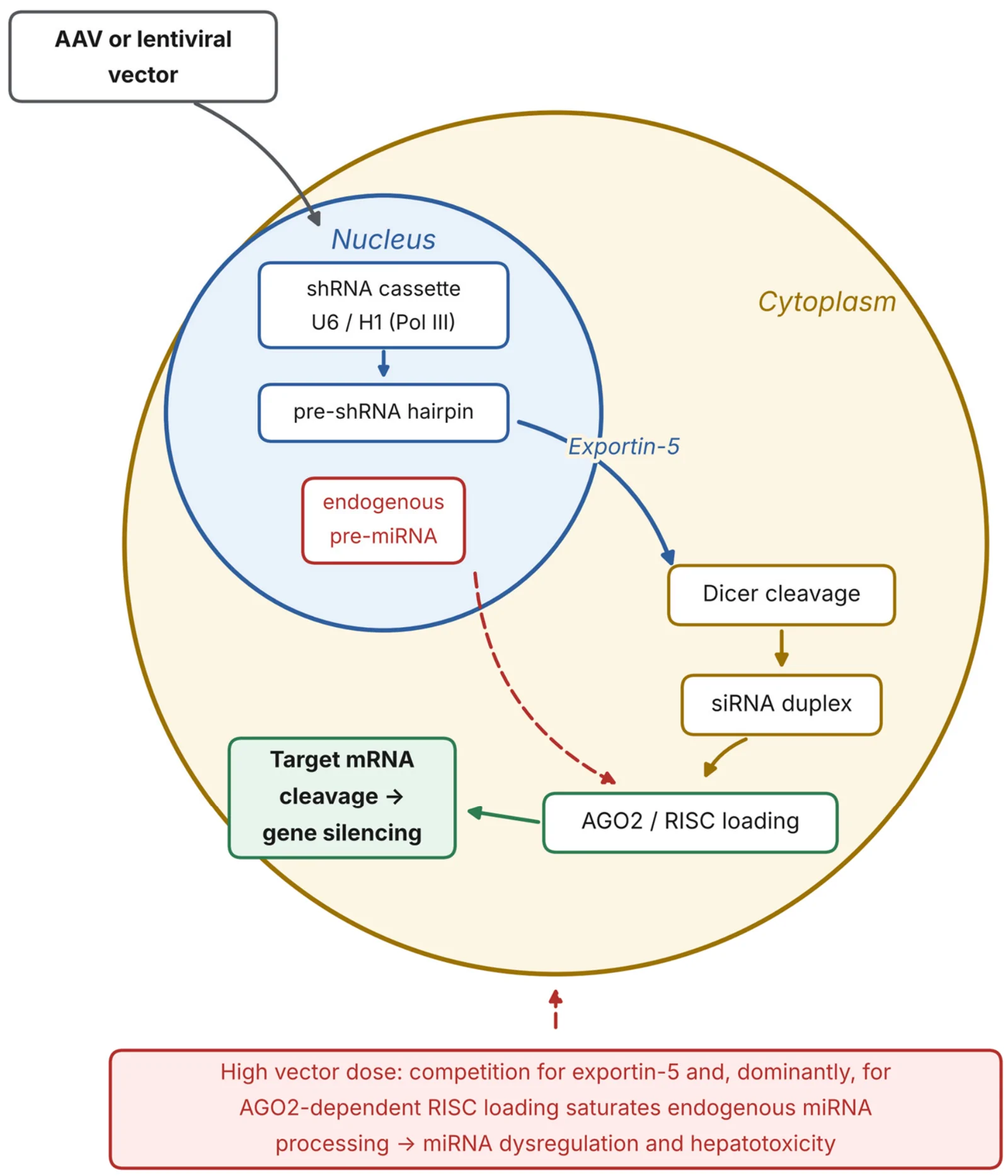
Mechanism of shRNA-mediated gene silencing. An shRNA expression cassette delivered by a viral vector (AAV or lentivirus) is transcribed in the nucleus by RNA polymerase III, exported to the cytoplasm by exportin-5, processed by Dicer into a mature siRNA duplex, and loaded into the RNA-induced silencing complex (RISC) to direct Argonaute2 (AGO2)-mediated cleavage of the target mRNA. At high vector doses, the pre-shRNA competes with endogenous pre-microRNAs (pre-miRNAs) for the same processing machinery, which can saturate the pathway and produce hepatotoxicity; this competition occurs both at the exportin-5-dependent nuclear export step and, dominantly, at Argonaute-2-dependent RISC loading (Section 8.2). The cell is drawn as a single schematic cell in which the nucleus is a small circle contained within the larger circle representing the cytoplasm. Solid arrows indicate the sequential steps of vector entry, shRNA transcription, nuclear export, Dicer processing, RISC loading and target mRNA cleavage; dashed red arrows indicate competition of endogenous pre-miRNAs and of the high vector dose for the same processing machinery, which saturates the pathway and leads to miRNA dysregulation and hepatotoxicity. The cell types and gene targets are presented separately, and in more detail, in Section 7.

Following intracellular transcription, the shRNA precursor is exported from the nucleus by exportin-5 and cleaved by the RNase III enzyme Dicer into a mature ~21-nucleotide siRNA duplex, one strand of which (the guide strand) is loaded into the RNA-induced silencing complex (RISC) and directs Argonaute2-mediated cleavage of complementary target mRNA, functionally converging on the same effector machinery used by endogenous miRNAs and by synthetic siRNA (Figure 2) [4,5,6,7]. This shared machinery is simultaneously the source of shRNA’s principal advantage—durable, self-amplifying silencing from a single vector administration—and its principal liability, namely competition with endogenous miRNA biogenesis when shRNA is overexpressed, a phenomenon documented in a landmark study that reported fatal hepatotoxicity in mice receiving high-dose AAV-delivered shRNA due to saturation of the shared exportin-5/Argonaute-2 processing machinery, and consequent dysregulation of liver-derived miRNAs [8,9,10]; the dominant rate-limiting component of this machinery was subsequently localized to Argonaute-2-dependent RISC loading rather than to nuclear export (Section 8.2) [11]. Any assessment of the current status of shRNA in atherosclerosis therapy must therefore weigh a substantial and mechanistically coherent preclinical efficacy literature, summarized in this review and in a recent comprehensive survey of viral vector-based gene therapy approaches to atherosclerosis [12], against this class-associated safety liability and against the parallel, and in several respects more clinically mature, development of synthetic siRNA, antisense oligonucleotide, and gene-editing alternatives targeting the same genes.

This review is organized as follows. Section 1.1 defines the evidence-classification framework applied throughout. Section 2 summarizes shRNA vector design and the RNAi mechanism in more detail. Section 3, Section 4, Section 5 and Section 6 review shRNA studies targeting, respectively, hepatic lipid-metabolism genes, vascular inflammatory and inflammasome pathways, macrophage foam cell formation, and VSMC proliferation/restenosis. Section 7 places these shRNA-specific findings within the longer history of antisense oligonucleotide and local vascular gene-therapy approaches to restenosis and atherosclerotic inflammation, several of which reached human clinical trials well before shRNA vectors were developed. Section 8 addresses delivery vectors and toxicity. Section 9 places shRNA alongside competing RNA-based and gene-editing modalities that target the same genes. Section 10 summarizes the current clinical translation status, including, in Section Clinical Experience with Vector-Encoded shRNA Outside Atherosclerosis, the clinical experience with vector-encoded shRNA in indications outside atherosclerosis and what it implies for the risk–benefit case in atherosclerosis, and Section 11 and Section 12 discuss remaining challenges and conclusions.

### 1.1. Scope, Terminology, and Evidence Classification

To address the heterogeneity of the RNA-based and gene-silencing literature relevant to shRNA in atherosclerosis, this review explicitly distinguishes three tiers of evidence throughout. First, direct shRNA evidence comprises studies in which a DNA-encoded, vector-delivered short hairpin RNA cassette was itself the silencing agent tested; only this tier is treated as evidence for the shRNA modality specifically. Second, adjacent-modality evidence comprises studies using synthetic siRNA, antisense oligonucleotides, constitutive genetic knockout, or antimiR/antagomir approaches against the same or related targets; because these modalities differ mechanistically from vector-encoded shRNA in durability, reversibility, and delivery, such studies are cited only to establish target validity or mechanistic context and are not treated as evidence of shRNA efficacy or clinical maturity. Third, this review consistently separates direct atherosclerosis efficacy studies, typically performed in *ApoE*−/− or *LDLR*−/− hyperlipidemic mice, from restenosis and vascular-injury models, typically balloon- or wire-injury of the carotid or coronary artery, which are mechanistically related but model neointimal hyperplasia rather than spontaneous atherosclerotic plaque formation; evidence from the latter is described as technological or delivery precedent rather than as direct atherosclerosis therapeutic evidence. These three distinctions are applied explicitly in the tables and text of Section 3, Section 4, Section 5, Section 6 and Section 7, and in the overall conclusions of this review, and they directly qualify the scope of any statement made below about the maturity of shRNA as a therapeutic modality for atherosclerosis specifically.

### 1.2. Positioning and Core Innovation of This Review

Unlike prior reviews of RNA-based cardiovascular therapeutics, which typically treat gene-silencing modalities as an undifferentiated class, this review is organized around a single question: why has shRNA, despite offering a distinctive combination of durable, vector-encoded gene silencing that is non-permanent at the level of the target gene (although the vector itself cannot readily be withdrawn, Section 8.4), not translated clinically for atherosclerosis while mechanistically related modalities (synthetic siRNA, antisense oligonucleotides, CRISPR-based gene editing) have. Accordingly, the review is structured along a technical-advantage → preclinical-verification → technical-defect → optimization-strategy → technical-competition narrative arc: Section 2, Section 3, Section 4, Section 5 and Section 6 establish the mechanistic advantage of durable silencing and verify it against the target-specific preclinical evidence admissible under Section 1.1; Section Target Adaptability of shRNA Across Atherosclerosis-Relevant Genes explicitly infers, across these targets, which classes of genes are and are not well suited to shRNA intervention; Section 7 provides historical precedent, and Section 8 characterizes the technical and safety defects that have constrained translation, including saturation toxicity, tissue-specific vector behavior, immune responses, and integration-related risk, together with optimization strategies reported over the past three years; and Section 9 places shRNA in explicit, dimension-by-dimension competition with synthetic siRNA, antisense oligonucleotides, and CRISPR gene editing to identify the application niches in which shRNA’s specific combination of durability and gene-level reversibility (qualified by the non-withdrawable vector, Section 8.4) is not merely a theoretical property but a practically irreplaceable one. Because this question cannot be settled from the atherosclerosis literature alone, Section Clinical Experience with Vector-Encoded shRNA Outside Atherosclerosis additionally asks how vector-encoded shRNA has fared in those indications in which it has reached patients, which identifies the disease-specific risk–benefit threshold, rather than any intrinsic deficiency of the hairpin, as the operative barrier. This structure, rather than a gene-by-gene catalog, is the organizing principle and core innovation of the present review.

Figure 2 summarizes the shRNA processing pathway. The figure traces the shRNA-specific processing route—nuclear Pol III transcription, exportin-5-mediated export, Dicer cleavage, and RISC/AGO2 loading—and marks the point in this pathway, competition for exportin-5 and, principally, for Argonaute-2-dependent RISC loading at high vector dose, at which shRNA diverges mechanistically from both synthetic siRNA, which enters the pathway directly at the Dicer/RISC step without a nuclear export requirement, and antisense oligonucleotides, which act independently of the RNAi machinery altogether through RNase H-mediated or steric-blocking mechanisms; this divergence is the basis for the modality-specific off-target and saturation profiles compared in Section 9. The division of gene targets by cell type, shown separately in Section 7, anticipates the target-adaptability analysis developed in Section Target Adaptability of shRNA Across Atherosclerosis-Relevant Genes, where hepatocyte-restricted, dose-graded targets (PCSK9) are distinguished from dose-sensitive (ApoB) and vascular-wall-restricted (ANGPTL2, connexin 43) targets on mechanistic rather than purely anatomical grounds.

## 2. Mechanism of RNA Interference and shRNA Vector Design

### 2.1. From Double-Stranded RNA to Sequence-Specific Silencing

RNAi was first described as a phenomenon in which injection of double-stranded RNA into *Caenorhabditis elegans* produced potent and specific silencing of the homologous endogenous gene, far more effectively than injection of either single strand alone [3]. In mammalian cells, long double-stranded RNA activates the interferon response and non-specific translational shutdown; this limitation was circumvented by the discovery that chemically synthesized 21-nucleotide siRNA duplexes with characteristic 2-nucleotide 3′ overhangs could mediate sequence-specific silencing without triggering this innate immune response, establishing siRNA as a tractable reverse-genetics and therapeutic tool in mammalian systems [4].

### 2.2. shRNA Expression Cassettes

To convert transient, exogenously delivered siRNA into a durable, cell-autonomous silencing signal, several groups constructed DNA expression cassettes in which an RNA polymerase III promoter (most commonly U6 or H1) drives transcription of a single RNA that folds into a hairpin: a double-stranded stem corresponding to the target sequence and its reverse complement, connected by a short single-stranded loop [5,6,7]. Once transcribed, this shRNA precursor is exported to the cytoplasm by exportin-5 and cleaved by Dicer to release a mature siRNA duplex that is loaded into RISC, functionally reconstituting the same silencing event that would be produced by direct siRNA transfection but with the crucial difference that the cell continuously regenerates the trigger as long as the expression cassette is present and transcriptionally active [5,6]. This design allows shRNA cassettes to be packaged into integrating (lentiviral) or non-integrating but long-lived episomal (AAV) viral vectors, producing silencing that can persist for months to years after a single administration, in contrast to synthetic siRNA, whose effect typically lasts days to a few weeks in non-hepatic, non-chemically modified formulations [5,6,7,13].

### 2.3. Design Considerations and Off-Target Effects

Effective shRNA design requires selection of a target sequence with minimal homology to unrelated transcripts (to avoid off-target silencing via partial complementarity, particularly through the seed region that dominates miRNA-like off-target binding), avoidance of extreme GC content or long homopolymer stretches that impair Pol III transcription, and, for cross-species preclinical-to-clinical translatability, selection of sequences conserved between the preclinical species and humans, an approach explicitly used in the development of a cross-species *PCSK9* shRNA construct for atherosclerosis [14]. Because shRNA is processed through the same Dicer/RISC/Argonaute pathway used by the hundreds of endogenous miRNAs that regulate normal hepatocyte and vascular cell gene expression, the dose of shRNA expressed per cell—governed jointly by vector copy number, promoter strength, and hairpin processing efficiency—is now recognized as the single most important design parameter determining whether a given construct will silence its target safely or instead saturate the endogenous pathway and produce toxicity, a point elaborated further in Section 8 [8,9].

## 3. shRNA Targeting Hepatic Lipid-Metabolism Genes

### 3.1. PCSK9

PCSK9 is secreted predominantly by hepatocytes and promotes lysosomal degradation of the hepatic LDL receptor (LDLR), thereby raising circulating LDL cholesterol; monoclonal antibodies and, more recently, a synthetic siRNA (inclisiran) that block or silence PCSK9 are approved lipid-lowering therapies, which have made PCSK9 the most extensively studied shRNA target in atherosclerosis. Lentivirus-delivered *PCSK9* shRNA administered systemically to hyperlipidemic apolipoprotein E-deficient (*ApoE*−/−) mice reduced aortic atherosclerotic plaque area and lowered plaque macrophage content, an effect accompanied by reduced expression of tumor necrosis factor-α (TNF-α), interleukin-1β (IL-1β), monocyte chemoattractant protein-1 (MCP-1), Toll-like receptor 4 (TLR4) and nuclear factor-κB (NF-κB) in the aorta, demonstrating that *PCSK9* silencing attenuates atherosclerosis at least in part through suppression of TLR4/NF-κB-driven vascular inflammation rather than through LDL-receptor-dependent lipid lowering alone [15]. A more recent study engineered a chimeric AAV8 vector expressing a cross-species *PCSK9* shRNA (AAV8.P-*PCSK9* shRNA) that achieved approximately 90% hepatic transduction, silenced hepatic *PCSK9* for up to a year, lowered serum cholesterol and triglycerides while raising *LDLR* expression without hepatotoxicity in wild-type mice, and, in *ApoE*−/− mice, reduced atherosclerotic plaque area, increased markers of plaque stability, and decreased inflammatory macrophage infiltration, providing among the most complete preclinical efficacy and safety packages assembled to date for an shRNA construct in this disease area [14]. These in vivo shRNA data are consistent with mechanistic work showing that PCSK9 engages the LOX-1 scavenger receptor and TLR4/NF-κB signaling directly in vascular cells independently of LDLR [16], and with complementary siRNA (rather than vector-encoded shRNA) studies showing that *PCSK9* knockdown in cultured THP-1 macrophages and HUVECs suppresses oxidized LDL (oxLDL)-induced NF-κB activation and apoptosis, respectively [17,18], collectively supporting a dual lipid-lowering and direct anti-inflammatory rationale for *PCSK9* silencing that goes beyond the mechanism captured by antibody-based PCSK9 inhibition alone. Consistent with the evidence-classification framework introduced in Section 1.1, these siRNA findings [17,18] are treated as adjacent-modality mechanistic corroboration rather than as an expansion of the direct shRNA efficacy evidence for PCSK9, which rests specifically on the lentiviral and AAV8 shRNA studies described above [14,15].

### 3.2. Apolipoprotein B

Apolipoprotein B100 (ApoB) is the essential structural protein of LDL and very-low-density lipoprotein particles, and its hepatic silencing was among the earliest demonstrations that AAV-delivered shRNA could durably lower plasma cholesterol in vivo. In a foundational study, nineteen candidate shRNAs targeting conserved human, mouse and macaque ApoB sequences were screened, and a self-complementary AAV8 vector expressing the most effective candidate (shA10) produced dose-dependent hepatic *ApoB* knockdown, a corresponding reduction in total and LDL cholesterol in diet-induced dyslipidemic mice, and no overt toxicity at the doses tested, although, consistent with the known on-target phenotype of ApoB loss-of-function, hepatic lipid accumulation (steatosis) was observed as an expected mechanism-based side effect of markedly lowering ApoB-dependent VLDL export from the liver [13]. This finding illustrates a recurring theme in shRNA-based lipid-lowering strategies: because many hepatic lipid-metabolism genes have pleiotropic roles in lipoprotein assembly and export, sustained near-complete knockdown, which is precisely what a durable shRNA vector is designed to achieve, can convert a therapeutically desirable reduction in atherogenic lipoprotein secretion into an undesired hepatic lipid storage phenotype, underscoring the importance of tunable rather than maximal knockdown for chronic use. The reported absence of overt toxicity in this study [13] should, however, be read together with the subsequent analysis by the same group, in which livers treated with AAV-shApoB contained approximately twofold more proliferating hepatocytes at eight weeks than livers treated with an equivalent miR-30-embedded construct (AAV-miApoB) or with control vector, and showed upregulation of inflammation-associated genes and of the immediate-early transcription factors Fos and Jun, whereas the miRNA-scaffolded construct produced neither effect; apoptosis was not increased in any group, and no predicted off-target transcript was significantly altered by either construct [19]. The two reports are therefore complementary rather than contradictory: conventional serum and histological endpoints were indeed unremarkable at the doses tested, but a proliferative and transcriptional response detectable only by dedicated proliferation markers and liver transcriptome profiling was nonetheless present, and because dividing hepatocytes dilute episomal AAV genomes, this response plausibly also erodes the long-term durability on which the shRNA rationale depends. This same-target comparison is, to our knowledge, the earliest direct demonstration that embedding the guide sequence in a miRNA scaffold rather than expressing a Pol III-driven hairpin reduces the hepatic burden of shRNA expression, a point developed further in Section 8.2.

### 3.3. Angiopoietin-like Proteins 2 and 4

Angiopoietin-like protein 4 (ANGPTL4) and angiopoietin-like protein 2 (ANGPTL2) regulate, respectively, systemic triglyceride metabolism via inhibition of lipoprotein lipase and vascular inflammation and endothelial senescence. Although the pivotal ANGPTL4 study used an antisense oligonucleotide rather than an shRNA vector, it is instructive for the shRNA field because liver-targeted *ANGPTL4* silencing attenuated both hyperlipidemia and atherosclerosis development in APOE*3-Leiden.CETP mice, a humanized lipoprotein-metabolism model, confirming ANGPTL4 as a validated silencing target upstream of the same lipoprotein-lipase pathway that shRNA-based strategies could equally engage [20]. In a more direct precedent for vascular-wall shRNA delivery, a single intravascular administration of AAV serotype 1 (AAV1) encoding shRNA against *Angptl2* in atherosclerotic *LDLR*−/−, human ApoB100-transgenic mice reduced native aortic endothelial *Angptl2* expression by approximately 80%, reduced expression of the senescence marker p21 together with endothelial *Icam-1*, *Il-1β* and *Mcp-1*, triggered apoptotic clearance of senescent endothelial cells, and slowed aortic atheroma progression by 58% over three months, providing one of the clearest demonstrations to date that vascular-wall-targeted, rather than purely hepatic, shRNA delivery can durably modify atherogenesis through elimination of a pathogenic, senescent endothelial cell subpopulation [21]. As already noted above, the ANGPTL4 finding [20] was generated with an antisense oligonucleotide rather than an shRNA vector and is cited here solely as adjacent-modality target validation, consistent with the framework in Section 1.1; it does not itself contribute to the direct shRNA efficacy evidence base, which for this gene family rests on the *ANGPTL2* shRNA study [21].

## 4. shRNA Targeting Vascular Inflammatory and Inflammasome Pathways

The NLRP3 inflammasome, a cytosolic multiprotein complex that senses cholesterol crystals and other danger signals to drive caspase-1-dependent maturation of IL-1β and IL-18, has been directly targeted by lentivirus-delivered shRNA in *ApoE*−/− mice, in which *NLRP3* gene silencing reduced plaque progression, decreased plaque macrophage and lipid content, increased plaque smooth muscle cell and collagen content, and lowered a composite plaque vulnerability index, providing direct causal evidence, independent of pharmacological NLRP3 inhibitors, that the inflammasome contributes mechanistically to both plaque growth and instability [22]. Building on this model, systemic *NLRP3* shRNA was subsequently used to demonstrate that hyperhomocysteinemia aggravates atherosclerosis in *ApoE*−/− mice specifically through NLRP3 inflammasome activation, since *NLRP3* knockdown abrogated the excess plaque burden, macrophage infiltration and IL-1β/IL-18 elevation otherwise seen in hyperhomocysteinemic animals, thereby using shRNA as a mechanistic loss-of-function tool to dissect a specific pro-atherogenic risk factor pathway rather than solely as a therapeutic candidate in its own right [23]. A related upstream regulator of NLRP3 activation, the purinergic receptor P2X7R, was itself silenced by RNA interference in cultured macrophages and in *ApoE*−/− mice, where *P2X7R* knockdown suppressed oxLDL-induced NLRP3 inflammasome activation via inhibition of protein kinase R phosphorylation and attenuated atherosclerosis development in vivo, extending the inflammasome-silencing rationale one step further upstream in the signaling cascade [24].

Beyond the inflammasome, lentivirus-delivered shRNA against tumor necrosis factor-α converting enzyme (TACE/ADAM17), the sheddase responsible for generating soluble TNF-α, was injected directly into abdominal aortic plaques of rabbits, where *TACE* silencing reduced plaque burden and the vascular remodeling index, decreased macrophage and lipid content while increasing smooth muscle cell and collagen content, suppressed local expression of NF-κB subunit p65, inducible nitric oxide synthase, intercellular adhesion molecule-1 (ICAM-1), matrix metalloproteinase (MMP)-2 and MMP-9 activity, and increased transforming growth factor-β1, collectively enhancing plaque stability through a coordinated anti-inflammatory and pro-fibrotic shift in plaque composition, and illustrating that direct intraplaque (rather than systemic) shRNA delivery is technically feasible in a large-animal atherosclerosis model [25]. In a further example of RNAi-based silencing of an inflammation-relevant non-coding RNA, knockdown of the long non-coding RNA AK136714, which is elevated in human atherosclerotic plaque and plasma and stabilizes TNF-α, IL-1β and IL-6 transcripts through interaction with the RNA-binding protein HuR, protected the endothelial barrier, suppressed endothelial inflammation and apoptosis in vitro, and inhibited atherosclerosis formation in *ApoE*−/− mice, indicating that RNAi-based silencing strategies developed for shRNA against protein-coding genes are equally applicable to pathogenic long non-coding RNAs that amplify vascular inflammation post-transcriptionally [26].

## 5. shRNA and Related RNA Interference Approaches Targeting Macrophage Foam Cell Formation

Macrophage uptake of modified LDL through the scavenger receptor CD36 is a rate-limiting step in foam cell formation, the histological hallmark of the early atherosclerotic lesion. CD36 was shown to be required for oxLDL- and oxidized-phospholipid-induced macrophage lipid accumulation using *CD36*-null macrophages and specific oxidized phospholipid ligands, establishing the receptor, together with its downstream Lyn/MEKK2/JNK signaling cascade, as necessary for foam cell formation in vitro and in hyperlipidemic mice in vivo, and thereby defining CD36 as a rational candidate for RNAi-based knockdown even though the pivotal loss-of-function experiments in this line of work used genetic deletion rather than an shRNA vector [27,28]. A parallel line of investigation has targeted the lipid-sensing microRNA miR-33, an intronic miRNA co-transcribed with the gene encoding sterol regulatory element-binding protein-2, which represses ABCA1 and ABCG1, the transporters responsible for macrophage cholesterol efflux to apolipoprotein A-I and HDL; genetic or antisense-mediated inhibition of *miR-33* raises macrophage cholesterol efflux capacity and plasma HDL cholesterol, providing the mechanistic foundation for miR-33 inhibition, conceptually the inverse operation to shRNA-mediated gene silencing, as a complementary RNA-based strategy to reduce foam cell lipid burden [29,30]. Because both the CD36 uptake pathway and the miR-33/ABCA1-ABCG1 efflux pathway converge on the same net cholesterol balance within the macrophage, they illustrate that RNAi-based intervention in foam cell biology can in principle be deployed either to silence a pro-atherogenic uptake gene (a conventional shRNA knockdown strategy) or to de-repress an anti-atherogenic efflux pathway (an antagomir or “silencing-of-a-silencer” strategy), a distinction of practical importance when selecting shRNA versus antimiR chemistries for a given macrophage-directed target. Neither the CD36 knockout studies [27,28] nor the miR-33 genetic/antisense studies [29,30] involve a vector-encoded shRNA construct; per Section 1.1, they are presented here strictly as mechanistic target-validation evidence for macrophage foam-cell biology and are excluded from any claim regarding shRNA-specific preclinical maturity.

## 6. shRNA Targeting Vascular Smooth Muscle Cell Proliferation and Restenosis

Excessive VSMC proliferation and migration following endothelial denudation, whether during native atherosclerotic plaque progression or after percutaneous coronary or peripheral angioplasty, produces neointimal hyperplasia and restenosis, a distinct but mechanistically related therapeutic problem for which lentivirus-delivered shRNA has been extensively tested in balloon-injury models. Lentivirus-mediated shRNA knockdown of the gap junction protein connexin 43 (Cx43), whose expression is temporally dysregulated after balloon injury of rat carotid arteries, attenuated the development of vascular restenosis, implicating gap-junction-mediated intercellular communication between VSMCs as a modifiable driver of neointimal expansion [31]. In a complementary strategy aimed at selectively arresting VSMC proliferation without impairing the re-endothelialization required for vessel healing, a recombinant lentiviral vector using the smooth-muscle-restricted SM22α promoter to drive overexpression of the cell-cycle inhibitor p27 (rather than a conventional shRNA cassette) inhibited VSMC proliferation more effectively than paclitaxel, reduced the intima-to-media ratio, and, critically, did not compromise re-endothelialization of the injured carotid artery, illustrating a general design principle directly transferable to shRNA vectors, namely that cell type-restricted promoters can be used to confine RNAi-pathway engagement to VSMCs while sparing the overlying endothelium [32]. Taken together with the intraplaque *TACE* shRNA delivery described in Section 4 [25], these restenosis studies demonstrate that local, catheter- or vector-based delivery to the injured or diseased vessel wall is both feasible and, for VSMC-directed and macrophage-directed targets alike, mechanistically preferable to systemic hepatic delivery when the therapeutic objective is modification of local plaque or neointimal biology rather than systemic lipid lowering. In line with the model classification in Section 1.1, both the Cx43 [31] and SM22α-p27 [32] studies were performed in balloon-injury restenosis models rather than in spontaneous atherosclerosis models; their findings are reported here as evidence for shRNA vector feasibility and cell type-restricted delivery, not as direct evidence of anti-atherosclerotic efficacy.

Table 1 summarizes the representative shRNA studies discussed in Section 3, Section 4, Section 5 and Section 6, together with the vector used, the animal model, and the principal finding of each study.

### Target Adaptability of shRNA Across Atherosclerosis-Relevant Genes

The gene-by-gene evidence assembled in Section 3, Section 4, Section 5 and Section 6 permits an explicit inductive comparison of which classes of atherosclerosis-relevant genes are well suited to durable shRNA silencing and which are not, a distinction the preceding sections do not draw out directly. Targets for which sustained, near-maximal knockdown is itself the desired chronic phenotype—secreted, hepatocyte-restricted regulators such as PCSK9, and inflammasome or sheddase components such as NLRP3 and TACE/ADAM17 that operate through threshold-dependent activation—are the most favorable candidates for shRNA, because their pathogenic action is graded with target abundance and tolerates the multi-month to multi-year suppression that a vector-encoded hairpin provides. By contrast, targets whose loss-of-function carries a dose-sensitive, on-target liability, such as ApoB, for which sustained near-complete knockdown produces hepatic steatosis as an on-target side effect (Section 3.2), are mechanistically poor candidates for a maximal, durable shRNA cassette and are better matched to synthetic siRNA or antisense chemistries whose effect can be titrated or withdrawn. A second axis of adaptability is sequence and pathway context: targets that share extensive seed-region homology with abundant endogenous miRNAs, or whose physiological regulation already depends heavily on the Dicer/RISC/Argonaute machinery, are disproportionately exposed to the saturation toxicity described in Section 8.2 and to off-target silencing via partial seed complementarity (Section 2.3), making them less suitable for high-copy shRNA expression regardless of their disease relevance. A third axis is tissue tropism and promoter availability: genes whose primary site of pathogenic action is the vascular wall (ANGPTL2, connexin 43) are shRNA-adaptable only where a validated, sufficiently restrictive vascular promoter or vector serotype exists (Section 8.1) [33]; in the absence of such a tool, systemic delivery risks off-target hepatic exposure and RNAi-pathway competition without a compensating therapeutic benefit at the vessel wall. Targets lacking any of these three favorable properties—graded rather than dose-sensitive pathogenicity, low homology to abundant endogenous miRNAs, and an available cell type-restricted delivery route—are better addressed, at the current state of the art, by the alternative RNA-based and gene-editing modalities compared systematically in Section Comparison Across RNA-Based and Gene-Editing Modalities.

## 7. Historical Precedents: Antisense Oligonucleotides and Local Vascular Gene Therapy for Restenosis and Atherosclerotic Inflammation

Placing the shRNA-specific findings summarized in Section 3, Section 4, Section 5 and Section 6 in proper historical context requires recognizing that atherosclerosis and restenosis have, for more than three decades, been described within a broader paradigm in which lipid accumulation, chronic inflammation, and monocyte/macrophage recruitment jointly drive lesion initiation and progression [34,35,36,37]. Long before shRNA vectors were available, the causal contribution of specific adhesion molecules and chemokines to this process had already been established using constitutive gene knockout rather than RNA interference: VCAM-1 is selectively induced on the aortic endothelium at lesion-prone sites before macroscopic plaque is detectable [38], combined P-selectin/*ICAM-1* deficiency substantially protects hyperlipidemic mice from atherosclerosis [39], and hypomorphic VCAM-1 mutant mice confirmed a major, non-redundant role for VCAM-1, but not ICAM-1, specifically in early lesion initiation [40]. The chemokine axis now targeted indirectly by several RNAi and antisense strategies discussed above was validated in an analogous way: *CCR2*-deficient *ApoE*−/− mice show markedly reduced lesion formation [41], MCP-1-deficient mice on an atherogenic human *ApoB*-transgenic background are similarly protected [42], and MCP-1 deficiency reduces atherosclerosis in LDL-receptor-deficient mice as well [43], while combined deficiency of the monocyte growth factor macrophage colony-stimulating factor and ApoE nearly abolishes lesion formation by preventing monocyte/macrophage accumulation in the vessel wall [44]. These knockout studies directly motivated the earliest gene-therapy (as opposed to genetic knockout) attempts to silence the same chemokine axis: an N-terminally truncated, dominant-negative MCP-1 mutant delivered by plasmid or adenoviral gene transfer attenuated atherosclerosis in *ApoE*-knockout mice [45] and suppressed neointimal hyperplasia after periarterial injury in mice and non-human primates [46], establishing monocyte chemoattractant protein-1 blockade as one of the first vascular-wall gene-therapy targets to be validated across both atherosclerosis and restenosis models and to be reviewed as a general anti-inflammatory strategy for vascular disease [47]. The scavenger receptor CD36, discussed in Section 5 as a candidate for RNAi-based knockdown, was itself established as the principal receptor mediating macrophage uptake of oxidized and nitrosated LDL using receptor-null macrophages [48] and shown to signal foam cell formation through a Vav-dependent, calcium- and dynamin-regulated internalization pathway [49], providing the mechanistic basis for *CD36* knockdown strategies.

An entirely parallel, and chronologically earlier, translational literature addressed VSMC-driven restenosis using antisense oligonucleotides and local gene delivery rather than shRNA, reflecting the fact that antisense chemistry matured as a deliverable therapeutic modality more than a decade before viral shRNA vectors were developed for vascular use, building on a general understanding of VSMC phenotypic plasticity in disease [50,51,52]. Antisense oligonucleotides against the proto-oncogene c-myb, delivered locally to injured rat carotid arteries, were shown in a landmark 1992 study to inhibit intimal arterial smooth muscle cell accumulation in vivo [53], and this was followed by intraluminal delivery of antisense oligonucleotides against *cdc2* kinase and proliferating cell nuclear antigen, which produced chronic inhibition of neointimal hyperplasia after a single local treatment [54]. These preclinical successes progressed to human trials substantially earlier than any shRNA construct: ex vivo treatment of human vein grafts with an *E2F* transcription factor decoy oligonucleotide reduced graft failure in the single-center PREVENT trial [55], although the larger, multicenter PREVENT IV trial of the same E2F decoy (edifoligide) in coronary artery bypass vein grafts failed to demonstrate efficacy on graft failure, underscoring how translational results obtained in small single-center studies of local vascular gene therapy have not always been reproduced at scale [56]. A conceptually related local antisense strategy, using oligonucleotides against *c-myc* delivered by intracoronary catheter at the time of stenting, was tested in the randomized ITALICS trial for prevention of in-stent restenosis [57]. These human trials were themselves the direct descendants of the foundational demonstration that direct gene transfer into the arterial wall could achieve site-specific transgene expression in vivo [58], a technical milestone without which none of the subsequent local antisense, shRNA, or gene-therapy strategies discussed in this review would have been conceivable.

Device-based local delivery has continued to evolve well beyond the catheter-based antisense trials described above, and several of these platforms are directly relevant to the future local shRNA delivery strategies discussed in Section 11: coronary stents embedded with siRNA against the pro-survival kinase Akt1 suppressed post-angioplasty restenosis in a rabbit model [59], DNA-eluting, controlled-release stents achieved sustained local transgene expression in porcine coronary arteries [60], and head-to-head comparison of adenoviral and AAV vectors delivered from stent surfaces demonstrated serotype-dependent differences in vascular gene transfer efficiency directly analogous to the AAV8-versus-AAV1 tropism differences exploited by the hepatic and vascular-wall shRNA studies reviewed in Section 3 and Section 4 [61]. Bisphosphonate-mediated conjugation chemistry has similarly been used to tether gene vectors directly to the metal surfaces of stents [62], and, in an earlier generation of device-based local pharmacotherapy that predates gene-based approaches entirely, angiopeptin-eluting stents were shown to reduce neointimal proliferation in a porcine coronary model [63], establishing local, device-based delivery as a therapeutic principle in vascular medicine well before its adaptation to nucleic acid payloads. Local blockade of vascular endothelial growth factor after intraluminal injury has likewise been shown to suppress experimental restenosis by inhibiting recruitment of monocyte-lineage cells to the injured vessel wall [64], reinforcing the theme, recurrent throughout this review, that restenosis and atherosclerotic inflammation share overlapping monocyte/macrophage-dependent mechanisms that can be interrupted at multiple molecular nodes by gene-silencing strategies, whether delivered by antisense oligonucleotide, shRNA, or siRNA-eluting device.

Table 2 summarizes these historical antisense oligonucleotide and local vascular gene-therapy precedents alongside the device platforms used to deliver them, situating the shRNA-specific literature reviewed in Section 3, Section 4, Section 5 and Section 6 within this longer translational trajectory.

**Figure 3:**
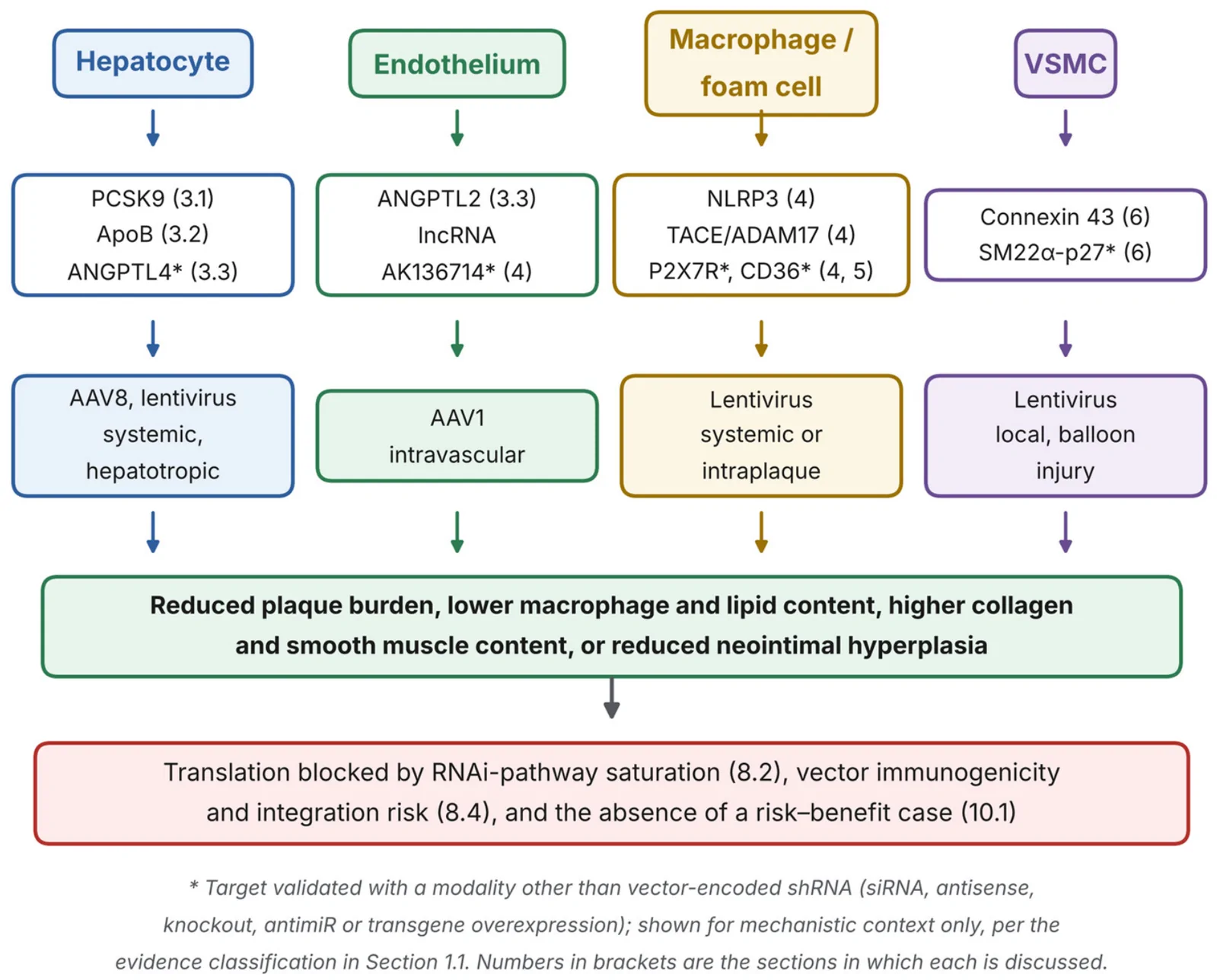
shRNA tissue targets, vectors and delivery routes across Section 3, Section 4, Section 5, Section 6 and Section 7. For each site of action—hepatocyte, endothelium, macrophage/foam cell and vascular smooth muscle cell (VSMC)—the figure lists the silenced targets, with the section in which each is discussed given in parentheses, the vector and delivery route used, and the preclinical outcome common to these studies. Targets marked with an asterisk were validated with a modality other than vector-encoded shRNA (synthetic siRNA, antisense oligonucleotide, genetic knockout, antimiR, or transgene overexpression) and are shown for mechanistic context only, in line with the evidence classification set out in Section 1.1. The lowest box records the three obstacles that have prevented clinical translation: saturation of the endogenous RNA interference pathway (Section 8.2), vector immunogenicity and integration-related risk (Section 8.4), and the absence of an acceptable risk–benefit case for atherosclerosis specifically (Section Clinical Experience with Vector-Encoded shRNA Outside Atherosclerosis).

Consistent with the model classification introduced in Section 1.1, none of the constitutive-knockout, antisense, decoy-oligonucleotide, or device-based studies summarized in Table 2 and discussed above constitutes direct evidence for shRNA-mediated silencing, and several were performed in restenosis or vascular-injury models rather than in spontaneous atherosclerosis models. This literature is presented here as historical and technological precedent—establishing that local vascular gene delivery, chemokine-axis silencing, and device-based nucleic acid delivery are each feasible—rather than as evidence that either shRNA specifically, or its therapeutic effect on atherosclerosis as opposed to restenosis, is already preclinically established. Figure 3 summarizes the tissue targets, vectors and delivery routes discussed across Section 3, Section 4, Section 5, Section 6 and Section 7, together with the evidence tier to which each belongs and the obstacles that have so far prevented translation.

## 8. Delivery Vectors, Biodistribution, and Toxicity

### 8.1. Viral Vectors

Nearly all of the shRNA studies reviewed in Section 3, Section 4, Section 5 and Section 6 used either lentiviral or AAV vectors, reflecting the field’s general convergence on integrating or long-lived episomal viral delivery as the only practical means of achieving the multi-month to multi-year duration of gene silencing that a chronic disease such as atherosclerosis requires, a convergence also evident across the broader RNA-therapeutics delivery landscape reviewed elsewhere [65]. Lentiviral vectors, used for intraplaque TACE delivery in rabbits [25], for systemic PCSK9 [15] and NLRP3 [22,23] silencing in mice, and for *Cx43* silencing in a rat balloon-injury restenosis model [31], integrate into the host genome and therefore in principle confer lifelong expression in transduced cells and their progeny, but this same property raises a long-recognized, if to date clinically unrealized in the cardiovascular gene-therapy context, theoretical risk of insertional mutagenesis. AAV vectors, used for hepatic *ApoB* [13] and PCSK9 [14] silencing and for vascular-wall *Angptl2* delivery [21], persist predominantly as non-integrating episomes, achieve serotype-dependent tissue tropism (AAV8 for liver, AAV1 for vascular endothelium in the studies reviewed here), and have an established record of long-term transgene expression, in one *PCSK9* shRNA study documented for up to one year after a single injection [14], without demonstrated hepatotoxicity at the doses used.

### 8.2. Saturation of the Endogenous microRNA Pathway

The single most consequential toxicity finding in the shRNA field remains the 2006 report that a substantial fraction (23 of 49) of AAV-delivered shRNA vectors tested caused fatal hepatotoxicity in mice within a month of high-dose intravenous injection, and that morbidity correlated with high hepatic shRNA expression and consequent downregulation of endogenous liver-derived miRNAs, implicating competitive saturation of the shared exportin-5/Dicer/RISC processing machinery rather than any sequence-specific off-target effect of the individual shRNAs tested [8]. Critically, the same study found that an approximately ten-fold reduction in vector dose abolished the lethal phenotype while preserving therapeutic knockdown, establishing dose, rather than sequence design alone, as the dominant safety-determining variable for AAV-shRNA vectors [8,9]. This mechanism has since been corroborated in other species and delivery contexts, including early postnatal lethality in shRNA-transgenic pigs associated with dysregulation of hepatic miRNA-processing enzymes [66], reinforcing that saturation toxicity is a generalizable property of the shRNA modality rather than an artifact confined to a single vector, promoter, or species, and is consistent with earlier observations that DNA constructs designed to produce shRNA in transgenic mice can themselves show early lethality and an interferon response even before any AAV-based high-dose delivery is considered [10]. Practically, this body of work has reshaped shRNA vector design toward weaker or tissue-restricted promoters, lower vector doses titrated to the minimum effective knockdown rather than maximal knockdown, and, where feasible, embedding the shRNA guide sequence within an endogenous miRNA scaffold (a “shRNA-mimic” or miR-30-based design) so that it is processed through the natural Drosha/DGCR8 microprocessor pathway rather than relying solely on cytoplasmic Dicer processing of a Pol III transcript. Direct, same-target comparisons support this design change: artificial miRNA shuttles were less toxic than conventional shRNAs carrying an identical guide sequence both in cultured cells and in mouse brain and liver [67], the optimized miR-E backbone raises mature guide-strand yield sufficiently for effective silencing from a single integrated copy [68], and, for ApoB specifically, the miR-30-embedded construct avoided the hepatocyte proliferation and inflammatory transcriptional response produced by the Pol III-driven hairpin against the same target (Section 3.2) [19]. Combining such a scaffold with an endothelium-restricted promoter—for example the *Cdh5* (VE-cadherin) promoter, whose endothelial specificity is well established from lineage-tracing and conditional-deletion tools [69]—would in principle restrict both cell-type expression and per-cell processing burden for vascular-wall targets. We are not aware, however, of any published report of such a combined *Cdh5*-driven, miR-30-scaffolded shRNA construct applied to atherosclerosis, and it is therefore presented here, and in Section 11, as a proposed design rather than as an existing one. Mechanistically, this saturation reflects direct stoichiometric competition: exportin-5 and the Dicer/TRBP/Argonaute2-RISC-loading complex are present at a fixed, finite cellular copy number, so that when a high-copy-number shRNA expression cassette floods this pathway with pre-shRNA substrate, both the nuclear export step and the cytoplasmic RISC-loading step become rate-limited for endogenous pre-miRNAs, which are consequently underprocessed and depleted, dysregulating the hundreds of transcripts they normally repress. That both steps are genuinely saturable, rather than merely inferred to be so, is established by gain-of-function experiments. Overexpression of exportin-5 alone enhances silencing by both shRNAs and miRNAs, identifying nuclear export as a rate-limiting step [70]. The more informative subsequent analysis, from the group that first described AAV-shRNA hepatotoxicity, showed that Argonaute-2 is the dominant rate-limiting determinant downstream of export: Ago2 co-expression increased shRNA potency by up to ten-fold, and co-expression of exportin-5 together with Ago2 simultaneously raised silencing efficiency, lowered serum alanine aminotransferase and extended knockdown by approximately three months, whereas overexpression of exportin-5 on its own increased efficacy but also hepatotoxicity and mortality [11]. The formulation used in the earlier literature, and earlier in this section, which attributes saturation to an undifferentiated “exportin-5/RISC pathway”, therefore requires refinement: nuclear export is an early bottleneck, but the step whose relief most reduces toxicity is Argonaute-2-dependent RISC loading, and collective saturation of Argonaute-1 to Argonaute-4, rather than of exportin-5, is the more likely proximate cause of the lethal phenotype [11]. The practical consequences differ accordingly. A construct whose ceiling is set by Argonaute availability can be improved by raising per-copy processing efficiency or by lowering vector copy number, but not by interventions directed at nuclear export alone; and because Ago2 saturation was most pronounced for perfectly complementary trigger–target pairs, the therapeutically preferred fully matched guide design is also the design most exposed to it [11]. Promoter strength acts through the same mechanism: strong U6-driven expression was accompanied by progressive loss of vector genomes and of knockdown, whereas the weaker H1 and 7SK promoters sustained more than 90% target silencing for three and a half months to more than a year [11]. This competition is not uniform across tissues: hepatocytes, which express a smaller and less diverse endogenous miRNA repertoire dominated by a few highly abundant species such as miR-122, which alone accounts for a large majority of hepatic miRNA content [71], appear particularly vulnerable to lethal saturation at high AAV8-shRNA doses, whereas vascular endothelium and macrophages, which express a broader and more redundant miRNA repertoire, more often show sub-lethal dysregulation of specific miRNA-dependent pathways (for example, senescence or inflammatory signaling) rather than overt cytotoxicity at comparable vector doses, though direct, dose-matched comparisons across the three tissue types within a single study remain scarce, so that this tissue gradient should be read as a hypothesis consistent with the available single-tissue data rather than as an established quantitative ranking. Over the past three years, several additional refinements have been reported to further narrow this safety margin without sacrificing durability, including capsid engineering that increases cell type-restricted transduction efficiency and thereby permits a lower total vector dose for an equivalent tissue-level knockdown [33], and further truncation and stem-loop optimization of miR-30/miR-E-type shRNA-mimic scaffolds to increase per-copy processing efficiency, allowing a lower vector copy number to achieve the same steady-state guide-strand abundance [68].

### 8.3. Non-Viral Delivery and Formulation Toxicity

Non-viral shRNA delivery, using cationic lipid or polymer nanocarriers, has been pursued as an alternative to viral vectors, but cationic nanocarrier chemistries themselves have been shown to cause cell necrosis through impairment of Na+/K+-ATPase activity and consequent inflammatory activation, a formulation-intrinsic toxicity that is independent of, and additive to, the RNAi-pathway saturation toxicity described above, and that has more often been documented for synthetic siRNA and lipid-nanoparticle-formulated nucleic acids than for plasmid- or viral-vector-encoded shRNA specifically [72]. Taken together, the toxicological literature indicates that any shRNA construct intended for chronic cardiovascular use must be evaluated not only for on-target efficacy but for dose-dependent saturation of the endogenous miRNA pathway (an intrinsic property of the shRNA modality) and, where non-viral carriers are used, for carrier-intrinsic membrane and metabolic toxicity (a property of the delivery formulation rather than the RNAi payload).

### 8.4. Immune Responses to Viral Vectors and the Risk of Irreversible, Integration-Related Silencing

Beyond RNAi-pathway saturation and carrier-intrinsic toxicity, two additional, clinically consequential safety liabilities of viral shRNA delivery have received comparatively little attention in the atherosclerosis-focused shRNA literature reviewed above and warrant explicit discussion. First, both AAV and lentiviral capsids are immunogenic: pre-existing neutralizing antibodies against common AAV serotypes are prevalent in the human population and can neutralize vector before transduction, while capsid- and transgene-directed cytotoxic T-lymphocyte responses can eliminate transduced cells over time, a phenomenon documented across AAV gene-therapy programs generally and not specific to any single transgene [73]; for a chronic, decades-long disease such as atherosclerosis, in which redosing may eventually be needed as expression wanes or as a superior construct becomes available, this pre-existing and vector-induced immunity represents a structural barrier that dose-titratable synthetic siRNA and antisense platforms do not share. Second, although recombinant AAV vectors are predominantly episomal, low-frequency genomic integration has been documented for both AAV and lentiviral vectors, and, where it occurs near a proto-oncogene or tumor-suppressor locus, can in principle cause insertional mutagenesis; this risk is substantially higher for integrating lentiviral vectors, which have been associated with clonal expansion and, in a minority of non-cardiovascular clinical gene-therapy programs, hematologic malignancy, than for non-integrating AAV, for which oncogenic integration has been observed in tumor-prone neonatal rodent models but not confirmed in long-term large-animal or human follow-up to date [74]. Because a lentiviral shRNA cassette that has integrated into the host genome cannot be withdrawn or reversed in the way a synthetic oligonucleotide can simply be discontinued, this integration-related risk is conceptually distinct from, and additive to, the RNAi-pathway saturation toxicity discussed in Section 8.2, and it directly qualifies the reversibility that Section 9 and the Conclusions attribute to shRNA as a class: reversibility in the sense of biochemical, non-permanent target modification (in contrast to gene editing) does not equate to reversibility of the vector itself once genomic integration has occurred, a distinction that has not, to date, been systematically addressed in the atherosclerosis-specific shRNA literature reviewed here.

## 9. shRNA in the Context of Other RNA-Based and Gene-Editing Therapeutics

Because the disease processes silenced by shRNA in the studies reviewed above—leukocyte recruitment, inflammasome activation, and lipid accumulation—are themselves the central mechanistic themes of the broader atherosclerotic plaque inflammation literature [75], and because pharmacological anti-inflammatory strategies acting on the same pathways have been extensively reviewed elsewhere [76], it is useful to situate shRNA within the wider landscape of RNA-based and gene-editing tools that address these same targets by fundamentally different chemistries. Beyond the RNAi- and antisense-based strategies discussed above, VSMC-directed gene therapy has historically also targeted apoptosis regulators, given the established contribution of VSMC apoptosis to plaque remodeling and instability [77], angiogenic growth factor overexpression, despite evidence that some growth factors such as FGF-1 can themselves promote intimal hyperplasia when overexpressed in the arterial wall [78], and direct cell-cycle inhibition via adenoviral overexpression of the cyclin-dependent kinase inhibitor p21, which suppresses VSMC proliferation and neointima formation after balloon injury [79], while, at the level of basic VSMC biology, endothelial-derived Jagged1 has separately been shown to promote Notch3-dependent contractile differentiation of coronary artery VSMCs in coculture, illustrating a further, non-RNAi pathway through which VSMC phenotype might in principle be modulated therapeutically [80]. The clinical RNA-therapeutics landscape relevant to atherosclerosis has, in practice, been led not by shRNA but by chemically synthesized, GalNAc-conjugated siRNA and by antisense oligonucleotides, a broader evolution in oligonucleotide chemistry and delivery that has been comprehensively reviewed elsewhere [81], both of which avoid the vector-encoded, self-renewing property of shRNA (and therefore its associated saturation toxicity) at the cost of requiring repeated dosing. Inclisiran, a GalNAc-conjugated synthetic siRNA against hepatic *PCSK9*, produced sustained LDL cholesterol reductions of approximately 50% with twice-yearly subcutaneous dosing in the pivotal ORION phase 3 trials and is now approved for clinical use, making it, to date, the only RNAi-class therapeutic specifically developed for an atherosclerosis-relevant lipid target to reach the clinic [82]. Patisiran, a lipid-nanoparticle-formulated synthetic siRNA against transthyretin, was approved earlier for hereditary transthyretin amyloidosis and established the clinical and regulatory precedent for lipid-nanoparticle-delivered RNAi therapeutics more broadly, including the delivery chemistry subsequently adapted for GalNAc-siRNA conjugates such as inclisiran [83]. A parallel line of RNAi-based lipid-lowering work has targeted ANGPTL3, using GalNAc-conjugated siRNA that durably lowered triglycerides and, in preclinical models, reduced atherosclerosis in mice and non-human primates [84], and, in a mechanistically distinct approach, AAV-delivered CRISPR base editing that installed a permanent inactivating stop codon in hepatic Angptl3, achieving near-complete and, unlike RNAi, potentially permanent protein knockdown after a single treatment [85]. The same base-editing strategy has been applied directly to PCSK9, first as an in vivo proof-of-concept in mice [86] and subsequently as a durable, single-treatment cholesterol-lowering intervention in non-human primates [87], building on the broader CRISPR-Cas9 genome-engineering platform [88]. Both targets have since moved from preclinical to human testing, so that gene editing can no longer be described mainly as a preclinical approach. For PCSK9, an interim analysis of the phase 1 trial of VERVE-102, a lipid-nanoparticle-delivered adenine base editor, reported dose-dependent reductions in circulating PCSK9 (51% to 88%) and LDL cholesterol (9% to 62%) after a single infusion in 35 patients with heterozygous familial hypercholesterolemia and/or premature coronary artery disease, without dose-limiting toxicities and with reductions that appeared to be maintained in the participants who had reached one year of follow-up, although long-term safety, including off-target editing, remains to be established [89]. For ANGPTL3, a first-in-human phase 1 trial of CTX310, a lipid-nanoparticle-delivered CRISPR-Cas9 nuclease (rather than a base editor), showed dose-dependent reductions in ANGPTL3, triglycerides and LDL cholesterol [90], and one-year follow-up has since confirmed that these effects are durable: at the highest dose (0.8 mg/kg), mean reductions of approximately 79% in ANGPTL3, 48% in triglycerides and 53% in LDL cholesterol were sustained a year after a single infusion [91]. Relative to these alternatives, shRNA occupies a specific niche defined by its combination of durability (like gene editing, unlike synthetic siRNA) and, unlike gene editing, gene-level reversibility in principle (because it silences rather than permanently mutates the target gene, although the vector encoding it cannot readily be withdrawn once administered; Section 8.4), a combination that could in theory be advantageous for targets, such as ApoB, ANGPTL4, or NLRP3, whose complete and irreversible lifelong inactivation might carry on-target liabilities such as the hepatic steatosis observed with sustained *ApoB* knockdown [13], but this theoretical advantage has not yet been matched by clinical development investment or by resolution of the saturation-toxicity concerns discussed in Section 8, a gap that has widened as gene-editing therapies against PCSK9 and ANGPTL3 have now generated human clinical data [89,90,91].

### Comparison Across RNA-Based and Gene-Editing Modalities

To make the comparison summarized above explicit, Table 3 compares shRNA with the three competing modalities discussed in this section—synthetic siRNA, antisense oligonucleotides, and CRISPR gene editing—across six practically decisive dimensions: typical duration of effect, delivery vehicle and manufacturing difficulty, off-target and immunogenicity risk, reversibility, redosing feasibility, and current clinical status, drawing on the primary studies already cited in Section 8 and Section 9 together with recent comparative and delivery-focused literature [92,93].

This dimension-by-dimension comparison indicates that shRNA’s translational disadvantage is not a single deficiency but the additive combination of the highest delivery and manufacturing complexity, the most demanding immunogenicity profile, and, for lentiviral constructs specifically, an integration-related loss of reversibility (Section 8.4), none of which is offset, at the current stage of development, by a duration-of-effect advantage large enough to outweigh its costs relative to twice-yearly synthetic siRNA dosing. Table 3 also identifies the specific gap that would need to close for shRNA to become competitive: a delivery platform combining AAV-level durability with siRNA-level manufacturing simplicity and redosability, which, based on the vascular-targeted AAV engineering reviewed in Section 8.1 [33] and the nanoparticle-based nucleic acid delivery systems reviewed for atherosclerosis specifically [93], remains an active but unresolved area of development. At the same time, the human data now available for PCSK9 and ANGPTL3 gene editing [89,90,91] show that single-administration, durable lowering of the same targets is clinically achievable, which raises the bar that any durable shRNA construct must clear.

## 10. Current Clinical Translation Status and Challenges

Despite more than fifteen years of consistent preclinical efficacy signals across multiple gene targets, cell types, and delivery vectors, drawn from a heterogeneous mix of direct shRNA, adjacent-modality, and restenosis-model evidence as classified in Section 1.1, no shRNA construct has, to date, entered clinical development specifically for atherosclerosis or its risk factors, a translational gap that stands in contrast to the clinical maturity of synthetic siRNA (inclisiran) and antisense oligonucleotide (e.g., ANGPTL4-directed) approaches targeting the same or closely related genes, and to the broader landscape of non-antibody PCSK9-directed modalities that has developed around this single target over the past decade [20,82,94]. Several factors plausibly account for this gap. First, the saturation toxicity described in Section 8 [8,9,10] has made regulators and sponsors cautious about integrating or long-lived hepatic shRNA expression cassettes relative to dose-titratable, non-integrating synthetic oligonucleotides that can simply be withheld if adverse effects emerge. Second, viral vector manufacturing, immunogenicity (particularly pre-existing anti-AAV capsid immunity in the human population), and the practical difficulty of redosing an integrating or long-persisting vector if efficacy wanes or a superior target emerges are manufacturing and clinical-development hurdles that synthetic siRNA and antisense platforms, built on established oligonucleotide chemistry and manufacturing infrastructure, do not share to the same degree. Third, most of the preclinical shRNA literature reviewed in Section 3, Section 4, Section 5 and Section 6 was generated in *ApoE*−/− or *LDLR*−/− mice with follow-up periods of weeks to a few months, whereas human atherosclerosis develops over decades, so that the demonstrated durability of shRNA silencing, while genuinely differentiating relative to synthetic siRNA, has not yet been validated over a time-scale and in a species in which long-term, low-grade RNAi-pathway competition could plausibly manifest as subclinical hepatic or vascular toxicity not captured in existing rodent studies.

### Clinical Experience with Vector-Encoded shRNA Outside Atherosclerosis

The conclusion that shRNA has not translated requires a precise statement: it has not translated for atherosclerosis. Vector-encoded shRNA has in fact been administered to patients in other indications. Cal-1 (LVsh5/C46), a self-inactivating lentiviral vector encoding a short hairpin RNA against *CCR5* together with the membrane-anchored C46 fusion inhibitor, was taken through a full preclinical safety and efficacy package in human hematopoietic cells and humanized mice, including integration-site and in vitro immortalization analyses [95], and was subsequently evaluated in an adaptive phase 1/2 study in HIV-1-infected adults receiving autologous gene-modified CD4+ T cells and hematopoietic stem/progenitor cells (ClinicalTrials.gov identifier NCT01734850), with related protocols conducted at additional centers. In parallel, TT-034, an AAV8 vector expressing three shRNAs directed against conserved regions of the hepatitis C virus genome, produced dose-dependent hepatic shRNA expression without detectable toxicity and without evidence of endogenous miRNA pathway disruption for up to one year in non-human primates at doses spanning several orders of magnitude [96], and entered a first-in-human single-administration phase 1/2a study in chronic hepatitis C. Neither program yielded a marketed product—Cal-1 was not advanced beyond early-phase study, and the TT-034 program was overtaken by directly acting antiviral agents that cure hepatitis C with a short oral course—but both establish that the regulatory, manufacturing and clinical-monitoring path for a vector-encoded hairpin is navigable, and that the modality itself is not disqualifying.

Read against this cross-indication experience, the atherosclerosis-specific gap identified in this review is better described as the absence of an acceptable risk–benefit case than as a failure of the hairpin. The indications in which shRNA has reached patients share three features that atherosclerosis lacks. First, the target disease was either life-limiting (HIV-1 infection, in a population seeking alternatives to lifelong antiretroviral therapy) or associated with a defined and proximate organ endpoint (chronic hepatitis C progressing to cirrhosis), which tolerates the irreversibility and immunogenicity of a viral vector in a way that an asymptomatic, decades-long risk factor does not. Second, the comparator available at the time of development was imperfect, whereas any shRNA construct directed at PCSK9, ApoB or ANGPTL3 must now be weighed against statins, ezetimibe, monoclonal PCSK9 inhibitors and twice-yearly inclisiran, all of which are withdrawable and supported by cardiovascular outcome data [2,82]. Third, the treated population was small and closely monitored, whereas an atherosclerosis indication implies exposure of a very large and largely healthy primary-prevention population, in which even a low per-patient probability of RNAi-pathway saturation (Section 8.2) or of integration-related liabilities (Section 8.4) becomes an unacceptable aggregate burden. The practical corollary, developed in Section 11, is that the most plausible near-term atherosclerosis indication for shRNA is not primary prevention but a narrow, high-residual-risk niche in which the comparator is weak, the population is small and monitorable, and durability is itself the clinically decisive property.

## 11. Future Perspectives

Several developments could plausibly narrow the gap between the mechanistic promise of shRNA and its current lack of clinical translation in atherosclerosis. First, miRNA-scaffold (“shRNA-mimic”) designs that route the guide sequence through the natural Drosha/DGCR8 microprocessor pathway, together with weaker or inducible promoters and vector doses deliberately titrated below the saturation threshold identified by Grimm and colleagues, could substantially widen the safety margin without sacrificing durability [8,9]. Second, cell type-restricted promoters, exemplified by the SM22α-driven VSMC-restricted construct [32] and, as a design proposed in Section 8.2 rather than one already reported, by an endothelium-restricted *Cdh5*-driven, miR-30-scaffolded cassette [69], together with vascular-wall-restricted vector serotypes such as AAV1 [21], could confine shRNA expression, and therefore RNAi-pathway competition, to the specific cell population responsible for the pathogenic phenotype, reducing hepatic exposure for targets where the primary site of action is the vessel wall rather than the liver. Third, because inflammasome and inflammatory shRNA targets such as NLRP3 and TACE have shown some of the most consistent plaque-stabilizing effects across independent studies [22,23,25], intraplaque or catheter-based local delivery, already demonstrated in a large-animal model [25], may represent a more tractable near-term translational path than systemic hepatic delivery for these specific targets. Fourth, head-to-head comparison of shRNA against the clinically more advanced synthetic siRNA, antisense oligonucleotide, and gene-editing platforms reviewed in Section 9, ideally within the same target gene and animal model, would help clarify whether shRNA’s durability advantage justifies its added vector-related development complexity for any specific atherosclerosis-relevant target, or whether it is more prudently reserved for targets where gene-level reversibility, unavailable with permanent gene editing, is itself clinically desirable (bearing in mind that the vector itself cannot readily be withdrawn; Section 8.4). Fifth, combination regimens pairing cell type-restricted shRNA with existing small-molecule anti-inflammatory pharmacotherapy, such as PPARγ agonism, which has itself been shown to reduce vascular inflammation and stabilize plaques through macrophage polarization [97], may offer a more gradual, combinatorial route to plaque stabilization than either modality alone, and could allow lower, safer shRNA vector doses to be used. Finally, and in light of Section Clinical Experience with Vector-Encoded shRNA Outside Atherosclerosis, indication selection may matter more than any further incremental vector refinement: a construct developed first for a narrow, high-residual-risk or locally treatable population—homozygous familial hypercholesterolemia, or recurrent in-stent restenosis addressed by intraplaque or device-based delivery—would face a weaker comparator, a smaller and more closely monitored population, and a setting in which durability is itself the clinically decisive property, which is precisely the risk–benefit configuration that broad primary prevention denies it.

## 12. Conclusions

Short hairpin RNA technology has accumulated a substantial, but evidentially heterogeneous, preclinical literature relevant to atherosclerosis. Direct evidence generated with vector-encoded shRNA specifically demonstrates that durable RNA interference can silence hepatic lipid-metabolism genes (PCSK9, ApoB) and vascular inflammasome/inflammatory mediators (NLRP3, TACE) in atherosclerosis models, and a driver of VSMC proliferation (connexin 43) in restenosis models, producing reductions in atherosclerotic plaque burden or neointimal hyperplasia and favorable shifts in plaque or vessel-wall composition. As set out in Section 1.1, this shRNA-specific efficacy evidence should not be conflated with the larger body of mechanistic target-validation evidence generated using synthetic siRNA, antisense oligonucleotides, genetic knockout, or antimiR approaches (e.g., ANGPTL4, P2X7R, CD36, miR-33, lncRNA AK136714), which establishes the biological relevance of these targets but does not itself constitute evidence for the shRNA modality, nor with the restenosis and vascular-injury literature discussed in Section 6 and Section 7, which establishes technological and delivery precedent rather than direct atherosclerosis efficacy data. This preclinical efficacy is counterbalanced by a well-documented, mechanistically specific toxicity arising from saturation of the endogenous microRNA-processing pathway at high vector doses, and by the greater manufacturing, immunogenicity, and redosing complexity of viral vectors relative to the synthetic siRNA and antisense oligonucleotide platforms that have already reached clinical use for the same or overlapping gene targets, and, at the durable end of the spectrum, by gene-editing therapies against PCSK9 and ANGPTL3 that have now produced human phase 1 data [89,90,91]. Taken together, the current status of shRNA in atherosclerosis therapy is therefore best characterized as mechanistically promising and supported by a defined, still limited, set of direct preclinical efficacy studies, rather than as a preclinically mature modality in the broader, aggregate sense that the wider RNAi and gene-silencing literature might otherwise seem to imply: realizing its distinctive combination of durability and gene-level reversibility (qualified by the non-withdrawable vector; Section 8.4) will require vector and promoter engineering that deliberately operates below the RNAi-pathway saturation threshold, restricts expression to the specific hepatic, endothelial, macrophage, or smooth muscle cell population responsible for the pathogenic phenotype being targeted, and is validated by additional, target-matched shRNA-specific studies rather than by extrapolation from adjacent silencing modalities or injury models. Finally, the comparison with those indications in which vector-encoded shRNA has already been administered to patients (Section Clinical Experience with Vector-Encoded shRNA Outside Atherosclerosis) indicates that the answer to the question posed in the title is not that the hairpin failed, but that atherosclerosis has so far offered no clinical setting in which the durability of a vector-encoded hairpin outweighs its vector-level irreversibility, immunogenicity and manufacturing burden relative to withdrawable, outcome-validated alternatives; identifying such a setting is therefore as much a precondition for translation as any further vector engineering.

## Figures and Tables

**Table 1:** Representative shRNA studies targeting atherosclerosis-relevant genes. Rows using a modality other than vector-encoded shRNA (siRNA, unspecified-vector RNAi, or knockdown) are labeled accordingly in the Gene/RNA Target and Vector/Modality columns and are included for mechanistic context only, per the evidence classification in Section 1.1.

Gene/RNA Target	Vector/Modality	Model	Key Finding	Ref.
PCSK9	Lentivirus (shRNA)	*ApoE*−/− mice	Reduced plaque area and macrophage content; decreased TNF-α, IL-1β, MCP-1, TLR4, NF-κB in aorta	[15]
PCSK9	Chimeric AAV8 (shRNA)	Wild-type and *ApoE*−/− mice	~90% liver transduction; ≥1 year silencing; lowered cholesterol/triglycerides; reduced plaque area, increased plaque stability	[14]
Apolipoprotein B (ApoB)	Self-complementary AAV8 (shRNA)	Diet-induced dyslipidemic mice	Dose-dependent hepatic *ApoB* knockdown; reduced total and LDL cholesterol; hepatic lipid accumulation as on-target side effect	[13]
ANGPTL2	AAV1 (shRNA), vascular delivery	*LDLR*−/−, hApoB100+/+ mice	80% endothelial knockdown; reduced p21, *Icam-1*, *Il-1β*, *Mcp-1*; cleared senescent endothelial cells; 58% slower plaque progression	[21]
NLRP3	Lentivirus (shRNA)	*ApoE*−/− mice	Reduced plaque progression and vulnerability index; decreased macrophage/lipid content, increased collagen/SMC content	[22]
NLRP3	Systemic shRNA	*ApoE*−/− mice with hyperhomocysteinemia	Abrogated HHcy-aggravated plaque burden, macrophage infiltration, and IL-1β/IL-18 elevation	[23]
P2X7R (siRNA, non-shRNA)	siRNA/RNA interference	*ApoE*−/− mice; macrophages	Suppressed oxLDL-induced NLRP3 activation via PKR inhibition; attenuated atherosclerosis in vivo	[24]
TACE (ADAM17)	Lentivirus (shRNA), intraplaque injection	Rabbit aortic plaque model	Reduced plaque burden and remodeling index; decreased p65, iNOS, ICAM-1, MMP-2/9; increased TGF-β1 and collagen	[25]
lncRNA AK136714 (RNAi, non-shRNA)	RNA interference (knockdown)	*ApoE*−/− mice; HUVECs	Protected endothelial barrier; suppressed inflammation/apoptosis; inhibited atherosclerosis formation	[26]
Connexin 43 (Cx43)	Lentivirus (shRNA)	Rat carotid balloon-injury model	Attenuated development of vascular restenosis after balloon injury	[31]

Abbreviations: AAV, adeno-associated virus; ADAM17, a disintegrin and metalloproteinase 17; ANGPTL2, angiopoietin-like protein 2; ApoB, apolipoprotein B; ApoE, apolipoprotein E; Cx43, connexin 43; HHcy, hyperhomocysteinemia; HUVEC, human umbilical vein endothelial cell; ICAM-1, intercellular adhesion molecule 1; IL, interleukin; LDLR, low-density lipoprotein receptor; lncRNA, long non-coding RNA; MCP-1, monocyte chemoattractant protein-1; MMP, matrix metalloproteinase; NF-κB, nuclear factor kappa B; NLRP3, NOD-, LRR- and pyrin domain-containing protein 3; oxLDL, oxidized low-density lipoprotein; P2X7R, P2X purinoceptor 7; PCSK9, proprotein convertase subtilisin/kexin type 9; PKR, protein kinase R; RNAi, RNA interference; shRNA, short hairpin RNA; siRNA, small interfering RNA; SMC, smooth muscle cell; TACE, tumor necrosis factor-α converting enzyme; TGF-β, transforming growth factor beta; TLR4, Toll-like receptor 4; TNF-α, tumor necrosis factor alpha.

**Table 2:** Historical antisense oligonucleotide and local vascular gene-therapy precedents relevant to shRNA-based restenosis and anti-inflammatory strategies.

Approach/Target	Delivery Platform	Model/Trial	Key Finding	Ref.
Antisense c-myb	Local intraluminal oligonucleotide	Rat carotid balloon injury	Inhibited intimal arterial smooth muscle cell accumulation in vivo	[53]
Antisense cdc2 kinase/PCNA	Single intraluminal oligonucleotide delivery	Rat carotid balloon injury	Chronic inhibition of neointimal hyperplasia after a single local treatment	[54]
E2F decoy (edifoligide)	Ex vivo vein graft treatment	PREVENT trial (human, single-center)	Reduced vein graft failure in a single-center randomized trial	[55]
E2F decoy (edifoligide)	Ex vivo vein graft treatment	PREVENT IV trial (human, multicenter)	Failed to demonstrate efficacy on graft failure at larger multicenter scale	[56]
Antisense c-myc	Intracoronary catheter delivery at stenting	ITALICS trial (human)	Randomized trial of local antisense delivery for prevention of in-stent restenosis	[57]
Direct arterial gene transfer (foundational)	Direct injection into arterial wall	Porcine iliofemoral artery in vivo	First demonstration of site-specific transgene expression in the arterial wall in vivo	[58]
*Akt1* siRNA	siRNA-embedded coronary stent	Rabbit angioplasty model	Suppressed post-angioplasty restenosis	[59]
Reporter/therapeutic transgene	DNA controlled-release stent	Porcine coronary arteries	Sustained local transgene expression from stent surface	[60]
Reporter transgene (Ad vs. AAV)	Gene-eluting stent (adenoviral vs. AAV)	In vivo blood vessel wall	Serotype-dependent differences in vascular gene transfer efficiency from stent surface	[61]
Gene vector conjugation	Bisphosphonate-mediated stent-surface tethering	Preclinical vascular model	Enabled gene vector delivery directly from metal stent surfaces	[62]
Angiopeptin (non-genetic comparator)	Drug-eluting stent	Porcine coronary model	Reduced neointimal proliferation, establishing device-based local delivery as a therapeutic principle	[63]
VEGF blockade	Local delivery after intraluminal injury	Rodent restenosis model	Suppressed restenosis by inhibiting recruitment of monocyte-lineage cells	[64]

Abbreviations: Ad, adenoviral vector; AAV, adeno-associated virus; Akt1, RAC-alpha serine/threonine protein kinase; cdc2, cell division cycle 2 kinase; c-myb, MYB proto-oncogene transcription factor; c-myc, MYC proto-oncogene transcription factor; E2F, *E2F* transcription factor; ITALICS, Investigation by the Thoraxcenter of Antisense DNA using Local delivery and IVUS after Coronary Stenting; PCNA, proliferating cell nuclear antigen; PREVENT, Project of Ex vivo Vein graft Engineering via Transfection; siRNA, small interfering RNA; VEGF, vascular endothelial growth factor.

**Table 3:** Comparison of shRNA with competing RNA-based and gene-editing modalities relevant to atherosclerosis.

Modality	Typical Duration of Effect	Delivery Vehicle/Manufacturing Difficulty	Off-Target/Immunogenicity Risk	Reversibility	Redosing Feasibility	Current Clinical Status
shRNA (this review)	Months to years per single administration (vector-encoded, self-renewing) [5,6,7]	Integrating (lentivirus) or episomal (AAV) viral vector; complex, capsid-specific manufacturing	RNAi-pathway saturation (Section 8.2); AAV/lentiviral immunogenicity and, for lentivirus, integration risk (Section 8.4)	Gene-level silencing is non-permanent (no change in genomic sequence), but the vector is not readily withdrawn once administered and lentiviral vectors integrate (Section 8.4)	Constrained by pre-existing and vector-induced immunity [73]	No construct in clinical development for atherosclerosis (Section 10); phase 1/2 experience in other indications (Section Clinical Experience with Vector-Encoded shRNA Outside Atherosclerosis)
Synthetic siRNA (e.g., inclisiran)	Weeks to months per dose; twice-yearly dosing regimen [82]	GalNAc conjugate or lipid nanoparticle; established, non-viral manufacturing	Low immunogenicity; possible seed-region off-target silencing	Fully reversible; effect clears as the compound is degraded	Straightforward; designed for repeated dosing	Approved for hyperlipidemia (inclisiran) [82]
Antisense oligonucleotide (e.g., ANGPTL4-directed)	Weeks per dose	Chemically modified oligonucleotide, subcutaneous or systemic; mature manufacturing	Low immunogenicity; hybridization-dependent off-target risk	Fully reversible	Straightforward; established redosing regimens	Multiple ASOs in clinical development or use for lipid-related targets [20]
CRISPR gene editing (base editing and Cas9 nuclease editing)	Permanent after a single treatment [86,87]; durability documented for up to one year in humans (VERVE-102, CTX310) [89,91]	AAV- or lipid-nanoparticle-delivered editor; large payload, complex manufacturing	Genome-editing off-target risk; AAV immunogenicity when viral delivery is used	Not reversible (permanent DNA sequence change)	Not applicable; single-treatment intent	Human phase 1 data: PCSK9 (VERVE-102; dose-dependent PCSK9 and LDL-C lowering after a single infusion) [89] and ANGPTL3 (CTX310; ANGPTL3, triglyceride and LDL-C lowering sustained for one year) [90,91]; earlier non-human primate (PCSK9) and mouse (ANGPTL3) proof-of-concept [85,87]; no product approved

Abbreviations: AAV, adeno-associated virus; ANGPTL3, angiopoietin-like protein 3; ANGPTL4, angiopoietin-like protein 4; ASO, antisense oligonucleotide; CRISPR, clustered regularly interspaced short palindromic repeats; GalNAc, N-acetylgalactosamine; PCSK9, proprotein convertase subtilisin/kexin type 9; RNAi, RNA interference; shRNA, short hairpin RNA; siRNA, small interfering RNA.

## Data Availability

No new data were created or analyzed in this study. Data sharing is not applicable to this article.
